# Clinical significance of the hemodynamic gain index in patients undergoing exercise stress testing and coronary computed tomography angiography

**DOI:** 10.1186/s12872-023-03088-z

**Published:** 2023-02-03

**Authors:** Mohamad Jihad Mansour, Elie Chammas, Michael Winkler, Wael AlJaroudi

**Affiliations:** 1 Division of Cardiovascular Medicine, Clemenceau Medical Center Affiliated with Johns Hopkins International, PO Box 11-2555, Beirut, Lebanon; 2 Department of Cardiology, Université de Montréal, Montréal, QC Canada; 3 Department of Cardiovascular Medicine, Augusta University Medical Center, Medical College of Georgia, 1120 15th Street, Augusta, GA 30912 USA; 4 Department of Radiology, University of North Carolina at Chapel Hill, Chapel Hill, NC USA

**Keywords:** Coronary artery disease, Coronary computed tomography angiography, Exercise stress testing, Hemodynamic gain index

## Abstract

**Background:**

Many hemodynamic parameters provide limited information regarding obstructive coronary artery disease (CAD) during exercise stress testing particularly when exercise is suboptimal. Hemodynamic gain index (HGI) is a recent sensitive indicator of ischemia and has been associated with increased mortality. This study evaluated the clinical impact of HGI in patients who underwent concomitant exercise stress testing and coronary computed tomography angiography (CCTA).

**Methods:**

A total of 284 consecutive patients from the executive health program between 2010 and 2018 were identified. Resting and peak heart rate (HR) as well as systolic blood pressure (SBP) measurements were recorded. Framingham risk score (FRS), Duke treadmill score (DTS) and HGI $(\frac{{{\text{HRpeak}} \times {\text{SBPpeak}} - {\text{HRrest}} \times {\text{SBPrest}}}}{{{\text{HRrest}} \times {\text{SBPrest}}}})$ were calculated. The latter was divided into quartiles. CCTA was used as a reference test to detect any CAD. Multivariate analysis and artificial neural network were used to determine the independent predictors of obstructive CAD.

**Results:**

Mean age was 53 ± 12 years with 83% male. Mean HGI was 1.74 ± 0.67, with cut-off value of severely blunted HGI ≤ 1.25 (Quartile 4). Patients with severely blunted HGI were older, had higher FRS, and worse DTS. Patients with obstructive CAD had lower HGI when compared to those with normal CCTA/non-obstructive CAD (1.36 ± 0.53 vs. 1.77 ± 0.67, *P* = 0.005), and showed a higher prevalence of severely blunted HGI (44% vs. 22%, *P* = 0.019). After adjusting for traditional risk factors, HGI remained an independent predictor of obstructive CAD while severely blunted HGI was associated with threefold increased odds of having obstructive CAD (*P* = 0.05). Using artificial intelligence analysis, severely blunted HGI independently predicted obstructive CAD with an area under the curve of 0.83 and 0.96, and normalized importance of HGI of 100% and 63%, respectively for different models.

**Conclusions:**

Among patients who underwent concomitant exercise stress testing and CCTA, severely blunted HGI independently predicted obstructive CAD after multivariate adjustment for traditional risk factors.

**Supplementary Information:**

The online version contains supplementary material available at 10.1186/s12872-023-03088-z.

## Introduction

Despite recent advances in stress imaging, exercise testing hemodynamic responses remain essential metrics in the diagnosis of coronary artery disease (CAD). Chronotropic incompetence, hypotension and impaired heart rate (HR) recovery are well-known prognostic markers. The prognostic significance of hypertensive (HTN) response to exercise is however not well defined [1–3]. Although the Duke treadmill score (DTS) is generally effective for risk stratification, its prognostic value may be limited for patients with lower exercise tolerance [4]. Changes in ST-segment during exercise and/or recovery may sometimes be misleading, especially in women [5]. These hemodynamic changes reflect different physiologic attributes and provide only partial information on the clinical impact and decision-making strategy in light of a submaximal exercise.

A novel hemodynamic parameter from exercise stress testing, the hemodynamic gain index (HGI) was developed and has shown to be a strong predictor of all-cause mortality in both men and women, particularly when the cut-off value was < 1.1 and 1.27, respectively [6, 7]. While stress testing could be false negative, recent guidelines advocate more anatomical imaging in angina evaluation [8]. HGI takes systolic blood pressure (SBP) and HR response, two important factors together [6, 7]. Patients with CAD may not be able to exercise for a long time or increase HR or may have hemodynamic compromise due to ischemia. We hypothesize that severely blunted HGI response may reflect significant CAD. While published data did not correlate HGI with anatomical imaging, in the present study, we aimed to assess the association between HGI and obstructive CAD in patients with atypical chest pain.

## Methods

### Study population

A cohort of 302 consecutive outpatients was identified from an internationally recognized executive health program between January 2010 and July 2018 (described previously, 9–11. Patients with abnormal resting left ventricular ejection fraction, prior myocardial infarction, significant valvular disease, left bundle branch block, congenital heart disease, pericarditis, myocarditis, and previous cardiac surgeries, were excluded (n = 18), leaving 284 patients for the final analysis. All patients underwent upright bicycle exercise testing followed by same day CCTA.

Demographics and comorbidities were prospectively entered at the time of testing and were subsequently retrieved for analysis. Framingham 10-year risk score (FRS) for CAD was subsequently calculated [12, 13]. The study was approved by the Institutional Board Review committee and complied with the Declaration of Helsinki.

### Exercise stress testing and hemodynamics

Exercise testing was performed according to a multistage, variable load, upright bicycle ergometer starting by a workload of 25 W and increasing by an increment of 25 W for every 2 min as previously published [14]. Resting and peak stress heart rate (HR) and systolic blood pressure (SBP) measurements were recorded prospectively and subsequently retrieved from the database. There were 13 patients with missing hemodynamic data, but none had obstructive CAD. HGI was calculated as previously published [6],

${\text{HGI}} = \frac{{{\text{HRpeak}} \times {\text{SBPpeak}} - {\text{HRrest}} \times {\text{SBPrest}}}}{{{\text{HRrest}} \times {\text{SBPrest}}}}$, and divided into quartiles, with Q1 being the highest quartile (≥ 2.08, 75th percentile) and Q4 the lowest (≤ 1.25, 25th percentile). DTS was calculated using minutes of bicycle exercise time, ST changes on a 12-lead ECG, and angina score [15–17].

Exercise testing was terminated because of fatigue, leg cramps, dyspnea and exaggerated systolic BP response during exercise > 250 mmHg. There were no absolute indications noted to terminate exercise [18].

### Coronary computed tomographic angiography

CCTA was performed as a standard test on all patients for the diagnosis of CAD, using 64- slice GE Discovery 750 HD GSI scanner according to normative CCTA protocols as previously published [19]. All coronary scans were performed and interpreted by a level III CCTA certified cardiologist during the same day.

### Patient preparation and premedication

Resting HR was measured for all patients who underwent CCTA before the procedure with a target HR ≤ 60 bpm for optimal images. Accordingly, patients were prepared and often required the administration of intravenous metoprolol with or without ivabradine.

### Image acquisition, electrocardiogram gating, and coronary anatomy

The CCTA scan was an electrocardiogram (ECG)-gated retrospective dose modulated study performed similar to a previously published protocol [19]. The use of retrospective ECG gating enabled image reconstruction at different points of the RR interval, allowing the interpreting physician to choose the optimal phase for image interpretation. CCTA was considered normal, non-obstructive or abnormal/obstructive CAD (plaque with ≥ 70% stenosis in a major epicardial vessel or ≥ 50% stenosis in the left main coronary artery).

### Statistical analysis

Continuous data were expressed as mean ± standard deviation and compared using the two-tailed Student’s t-test for normally distributed data, and the Wilcoxon test for skewed data. Categorical data were displayed as frequencies and percentages, and compared using Pearson Chi-square test. To determine the incremental value of HGI, nested binary logistic modeling was performed: model 1 without HGI, and model 2 including HGI. The models were compared on the basis of difference in likelihood ratio and the resulting Chi-squared value looked up at one degree of freedom. In addition, multivariate regression analysis was performed to assess independent predictors of obstructive CAD. Given the relatively low number of obstructive CAD and to avoid overfitting of the model, we adjusted for FRS (which integrated age, gender, HTN and BP, dyslipidemia (DL) and smoking), diabetes, DTS (which included data on exercise time, electrocardiographic ischemic changes and angina symptoms) in addition to HGI. The latter was used as a continuous variable where quartiles 1 to 4 were compared using ANOVA test (for better graphical illustration, and to have a cut-off value for clinical use), as well as dichotomous comparing quartile 4 versus quartiles 1 to 3 (Q4 vs. Q1-3) using Student’s t-test for easier visualization and interpretation of the results. HGI (severely blunted ≤ 1.25 or > 1.25) was also compared against CCTA (obstructive or non-obstructive) using 2 × 2 table for all patients in order to calculate sensitivity (SN), specificity (SP), positive predictive value (PPV) and negative predictive value (NPV).

Predictors of obstructive CAD were also determined using machine learning artificial intelligence. Numerical data were entered in the artificial neural network model with multilayer perceptron. A 70/30 training/testing partition was used with 1 hidden layer. ROC curves were generated and independent variable important analysis was extracted. Two models were used; the first included FRS, diabetes, body mass index (BMI), DTS, and HGI; the other substituted FRS with age, gender, HTN, DL and smoking while keeping the other variables. All tests were 2-tailed, and a *P*-value < 0.05 (set a priori) was considered statistically significant. All statistical analyses were carried out with SPSS Statistics version 27 (IBM, Inc., Armonk, NY).

## Results

There were 284 patients (mean age 53 years, 83% male, and 17% diabetic) who had concomitant exercise stress testing and CCTA. The mean HGI was 1.74 ± 0.67 (Q1 ≥ 2.08, Q2 1.66–2.07, Q3 1.26–1.65, Q4 ≤ 1.25). Patients with severely blunted HGI ≤ 1.25 were older, had higher FRS, and worse DTS (Table 1).

**Table 1:** Demographics and clinical characteristics of the cohort stratified by hemodynamic gain index quartiles

	All (N = 271) (13 missing HGI/HR)	Q1 (N = 68)	Q2 (N = 68)	Q3 (N = 68)	Q4 (N = 67)	*P*-value
HGI		≥ 2.08	1.66–2.07	1.26–1.65	≤ 1.25	
HGI (mean ± SD)	1.74 ± 0.67	2.64 ± 0.53	1.85 ± 0.12	1.45 ± 0.12	1.00 ± 0.21	< 0.001
**Demographics**						
Age	52.9 ± 11.8	48.7 ± 10.9	52.9 ± 11.3	53.7 ± 10.7	56.5 ± 13.2	0.002
Male	225 (83%)	62 (91%)	59 (87%)	56 (82%)	48 (72%)	0.018
BMI (kg/m^2^)	29.0 ± 4.8	28.4 ± 4.6	29.1 ± 4.7	29.4 ± 4.5	29.4 ± 5.4	0.54
**Comorbidities**						
Hypertension	81 (30%)	18 (27%)	16 (23%)	23 (34%)	24 (36%)	0.34
Diabetes	45 (17%)	10 (15%)	10 (15%)	10 (15%)	15 (22%)	0.54
Dyslipidemia	82 (30%)	21 (30%)	19 (28%)	24 (35%)	18 (27%)	0.70
Smoking history	166 (61%)	42 (62%)	45 (66%)	47 (69%)	32 (48%)	0.055
Framingham Risk Score	24 ± 19	13 ± 15	19 ± 17	27 ± 18	35 ± 18	< 0.001
**Hemodynamics**						
Resting HR (bpm)	70 ± 10	62 ± 8	68 ± 9	72 ± 7	79 ± 10	< 0.001
Resting SBP(mmHg)	117 ± 12	113 ± 11	119 ± 10	119 ± 12	119 ± 13	0.003
Peak HR (bpm)	131 ± 20	148 ± 16	136 ± 19	124 ± 14	116 ± 14	< 0.001
Peak SBP (mmHg)	167 ± 17	170 ± 18	170 ± 16	168 ± 18	160 ± 14	< 0.001
% MPHR	84 ± 9	89 ± 7	85 ± 7	84 ± 7	77 ± 10	< 0.001
Exercise time (min)	9.4 ± 7.2	10.0 ± 4.6	9.3 ± 7.1	10.0 ± 9.1	8.0 ± 7.1	0.38
Duke treadmill score	3.5 ± 4.3	5.8 ± 4.6	3.9 ± 4.4	2.2 ± 3.8	1.6 ± 2.7	< 0.001

*BMI* body mass index, *bpm* beats per minute, *HGI* hemodynamic gain index, *HR* heart rate, *MPHR* maximum predicted heart rate, *SBP* systolic blood pressure

**Fig. 1:**
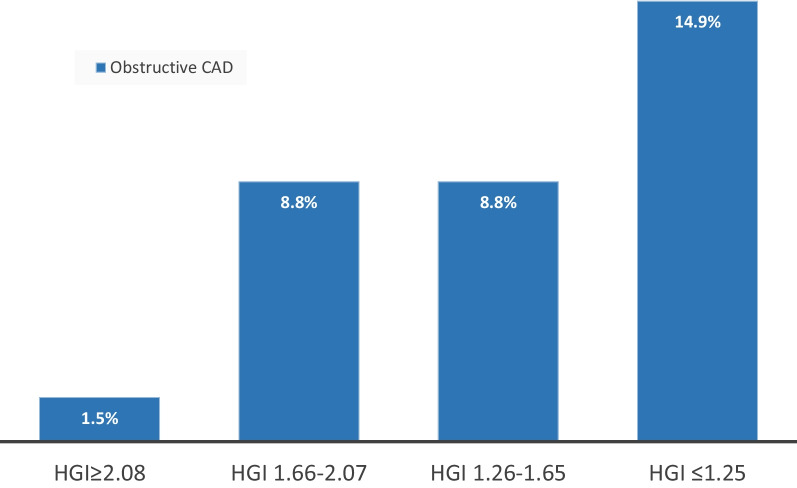
Prevalence of obstructive coronary artery disease stratified by quartiles of hemodynamic gain index

There were 23 (8%) patients with obstructive CAD on CCTA; they were older, had more co-morbidities particularly HTN and DL as compared to those without obstructive CAD (Table 2). Moreover, they showed lower HGI when compared to patients with normal CCTA or non-obstructive CAD (1.36 ± 0.53 vs. 1.77 ± 0.67, *P*-value 0.005), and had more prevalence of severely blunted HGI ≤ 1.25 (44% vs. 22%, *P*-value 0.019) (Table 2). The prevalence of obstructive CAD increased with progressive blunting of HGI (*P*-value 0.04) (Fig. 1).

**Table 2:** Baseline characteristics stratified by coronary computed tomography angiography results

	All (N = 284)	Obstructive CAD (N = 23)	Normal/non-obstructive (N = 261)	*P*-value
**Demographics**				
Age (years)	53 ± 12	68 ± 8	52 ± 11	< 0.0001
Male gender	235 (83%)	18 (78%)	217 (83%)	0.55
Body mass index (kg/m^2^)	29.0 ± 4.8	30.6 ± 6.4	28.9 ± 4.6	0.97
**Comorbidities**				
Hypertension	83 (29%)	16 (70%)	67 (26%)	< 0.0001
Diabetes	48 (17%)	7 (30%)	41 (16%)	0.071
Dyslipidemia	85 (30%)	16 (70%)	69 (26%)	< 0.0001
Smoking history	170 (60%)	15 (65%)	155 (59%)	0.58
Framingham Risk Score	24 ± 20	23 ± 14	25 ± 20	0.69
**Hemodynamics**				
Resting heart rate (bpm)	69 ± 10	72 ± 15	69 ± 9	0.16
Resting SBP (mmHg)	118 ± 12	124 ± 15	117 ± 11	0.010
Peak heart rate (bpm)	140 ± 18	143 ± 16	122 ± 20	< 0.0001
Peak SBP (mmHg)	167 ± 17	167 ± 18	167 ± 16	0.96
% MPHR	79 ± 11	81 ± 12	79 ± 11	0.38
Exercise time (min)	9.3 ± 7.3	7.6 ± 4.6	9.4 ± 7.4	0.32
Duke Treadmill Score	3.5 ± 4.3	2.1 ± 3.4	3.6 ± 4.4	0.16
Hemodynamic gain index	1.74 ± 0.66	1.36 ± 0.53	1.77 ± 0.67	0.005
Hemodynamic gain index ≤ 1.25	67 (24%)	10 (44%)	57 (22%)	0.019

*Bpm* beats per minute, *CAD* coronary artery disease, *MPHR* maximum predicted heart rate, *SBP* systolic blood pressure

Compared to CCTA, the SN, SP, PPV, and NPV of HGI as a predictor of obstructive CAD were respectively 43%, 78%, 15% and 83%.

Using nested binary logistic modeling, there was significant incremental value with HGI with resulting Chi-square added value 8.0 (*P*-value 0.005).

In the unadjusted model, HGI was associated with increased odds of obstructive CAD (HGI as continuous variable: odds ratio (OR) 0.29 [95% CI: 0.12–0.69], *P*-value 0.005), whereas severely blunted HGI ≤ 1.25 (Q4 vs. Q1-3) was associated with OR 2.75 (95% CI: 1.15–6.61), *P*-value 0.023. After adjusting for FRS, diabetes, DTS, HGI remained independent predictor of obstructive CAD (continuous variable OR 0.25 [95% CI 0.081–0.78], *P*-value 0.017). Severely blunted HGI ≤ 1.25 was associated with threefold increased odds of obstructive CAD (adjusted OR 3.02 [95% CI 1.01–9.11], *P*-value 0.05).

**Fig. 2:**
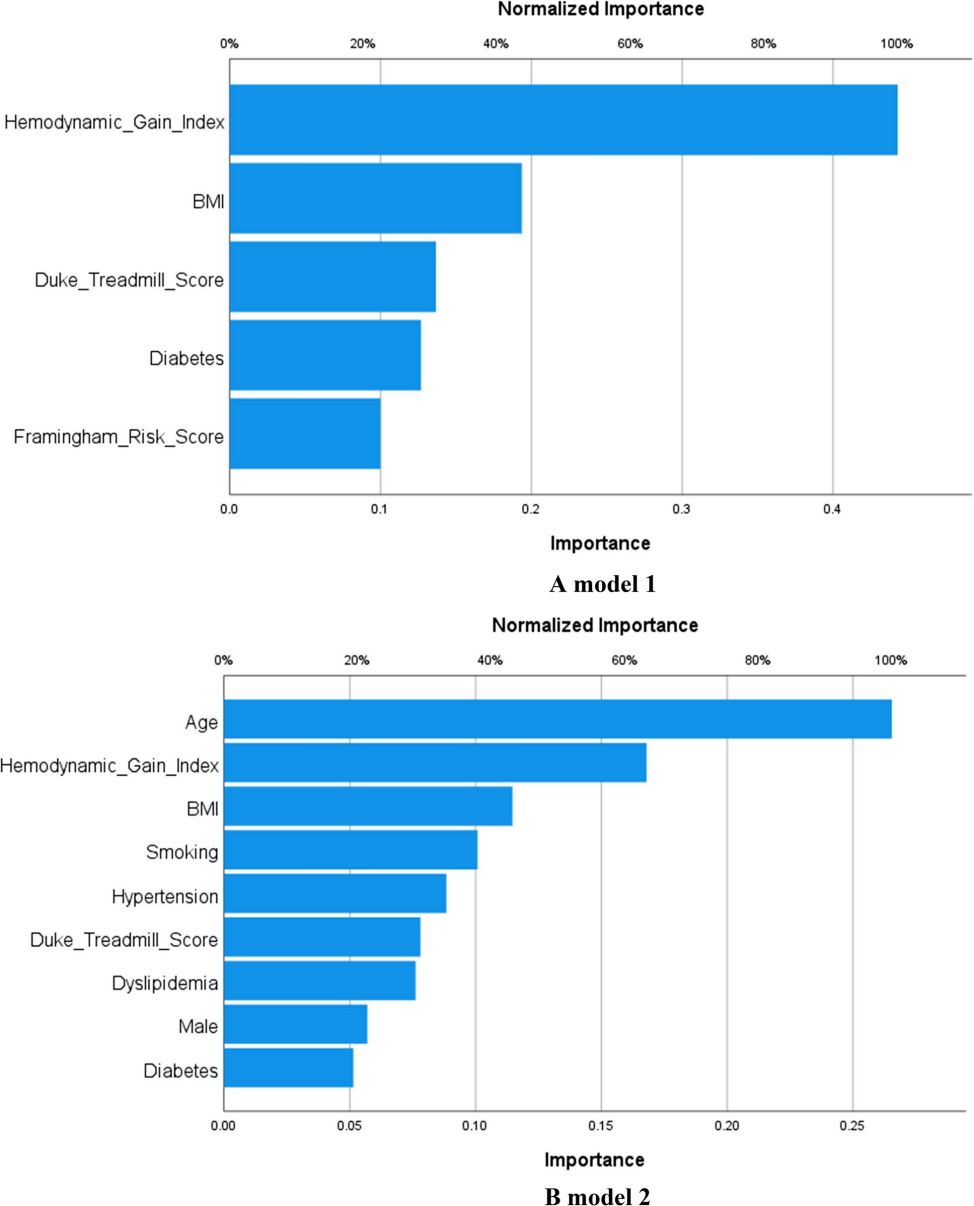
Machine learning model showing superiority of hemodynamic gain index over other variables with (**A**) and without (**B**) Framingham risk score, with an area under the curve of 0.83 and 0.96, respectively

Using machine learning artificial neural network, severely blunted HGI was an independent predictor of obstructive CAD on CCTA using models 1 and 2 (Additional file 1: Supplement Table 1) with an area under the curve of 0.83 and 0.96, and normalized importance of HGI of 100% and 63%, respectively (Fig. 2A, B).

## Discussion

With the present study, we aimed to assess the clinical significance of a newly developed hemodynamic index in patients with stable angina and atypical chest pain. As part of an executive screening program, exercise stress testing and CCTA were offered to patients at low-intermediate risk for CAD, as demonstrated by the FRS.

Although several classic hemodynamic markers have been associated with adverse cardiovascular outcomes, each has limitations [1, 2]. For instance, maximum predicted HR < 85%, hypotensive SBP response during exercise, and lower performance stratified by age and gender (as measured by metabolic equivalents of tasks) reflect different hemodynamic abnormalities and are measured at separate time points during the exercise test. These parameters reflect only a fractional hemodynamic response and lack the integration of both HR and SBP into one comprehensive metric. Nevertheless, the maximum HR-SBP product is an indirect measure of myocardial oxygen consumption and an alternative tool to these variables but is not always reliable to evaluate cardiac function and prognosis in patients with established cardiovascular disease [6].

Using HR and SBP responses to exercise testing, HGI was first developed by Vainshelboim and colleagues in 2019 on a large cohort of men and later validated on women [6, 7]. HGI utilizes the net gain (from rest to maximal exercise) in the rate-pressure product divided by resting values. This formula has a strong physiological rationale for assessing the responsiveness (net gain) in cardiovascular function. The authors found that higher HGI was inversely and independently associated with lower risk of all-cause mortality. HGI remained a strong predictor of mortality even after adjusting for multiple variables [6, 7]. In both studies however, the authors did not investigate for possible underlying CAD, but rather included patients with an established history of any cardiovascular disease. Our study is the first to establish a clinical strong correlation between HGI and obstructive CAD: while there was significant incremental value with HGI in predicting obstructive CAD using the nested binary logistic modeling, a severely blunted HGI ≤ 1.25 was a potential candidate for the prediction of obstructive CAD after adjusting for traditional risk factors. This is in fact significant because despite a submaximal exercise, a suboptimal/low HGI should raise suspicion for obstructive CAD even if the exercise testing is negative for ischemia. Indeed, the accuracy of exercise testing is limited, whether stress ECG or stress echocardiogram. It is no surprise that the updated guidelines now advocate anatomical imaging (particularly CCTA) in patients with no known CAD presenting with angina [8].

Compared to CCTA, HGI > 1.25 had a quite important NPV of 83% for excluding obstructive CAD while severely blunted HGI ≤ 1.25 had a SP of 78% for obstructive CAD. Compared to the standard stress ECG, HGI seems to be less sensitive but adds more specificity to the diagnostic testing [16]. Nevertheless, with only 1.5% of patients having obstructive CAD with a normal HGI ≥ 2.08 (Fig. 1), the mechanism behind this finding is not well defined. Perhaps the presence of collaterals despite obstructive CAD might play a role in exercise tolerance. In fact, HGI displays a physiologic role in exercise testing compared to CCTA that evaluates mostly the coronary anatomy. The use of fractional flow reserve-guided CCTA might have added value. In addition, we think that patients with collaterals, single versus multivessel disease could have higher HGI and this might be a future perspective and needs to be tested in larger cohorts.

Calculation of HGI is particularly important when patients might be referred for exercise testing in a non-cardiovascular setup (in-hospital setting or outpatient specialty clinic). The most common example might in fact refer to patients who undergo cardiopulmonary exercise testing (CPET) performed by pulmonologists [20]. In recent cohorts of patients who underwent CPET, HGI was independently associated with all-cause mortality in different groups of patients, namely those without and with established heart failure and CAD [21, 22].

Our study is the first to use HGI on bicycle stress test. Furthermore, we adjusted for the DTS which has also been validated with bicycle ergometer and shown to be a predictor of mortality [17]. In our cohort, HGI remained and independent predictor of obstructive CAD even after adjusting for DTS and traditional risk factors (Fig. 2A, B).

### Strengths and limitations

While published data provided a strong and independent association between HGI and mortality, [6, 7, 21, 22], to our knowledge, this is the first to show that severely blunted HGI may be a surrogate for obstructive CAD which in turn may explain the associated increased mortality. Our analysis suggests that severely blunted HGI cut-off value was comparable to previously published studies (1.25 vs. 1.1 and 1.27) [6, 7]. Moreover, we relied on nested binary logistic modeling, multivariate analysis and artificial intelligence to provide powerful statistical analysis. Artificial neural network in fact allows optimal modeling by automatically choosing non-linearities without a temptation to overfit.

Still, there are several limitations to our study. First, the number of obstructive CAD was relatively low, which is expected given the low-intermediate risk population, and this likely affected the NPV of HGI in ruling out obstructive CAD. Second, the cohort was predominantly of male gender as most executives were males. Gender however was not predictive of obstructive CAD (*P*-value 0.554). Third, this is a retrospective, single center study conducted on patients with low-intermediate risk, presenting with minimal symptoms or only for screening, and do not reflect other groups of patients presenting to clinic and daily practice. Fourth, we used CCTA as a gold standard to identify obstructive CAD rather than invasive coronary angiography or CCTA-fractional flow reserve. Still, patients with obstructive CAD on CCTA underwent subsequent coronary angiography. Of them, 8 had percutaneous coronary intervention and 2 had conservative medical therapy. Lastly, our data lacked medications history, namely beta-blockers and/or calcium channel blockers. Although confounding bias could be further exacerbated by medications that alter the HR-SBP product, their use did not affect HGI which was still a strong predictor of mortality as previously published [6, 7, 21, 22].

## Conclusions

In this retrospective single center study conducted on patients at low-intermediate FRS, severely blunted HGI ≤ 1.25 was an independent predictor of obstructive CAD on CCTA. HGI is a robust hemodynamic parameter that combines HR and BP response, easily obtained during exercise stress testing. HGI is practical and may be used to re-stratify patients, change management and provide prognostic information, therefore affecting clinical-decision making and outcome. Further validation of this parameter in larger multicenter databases is needed in the hope of integrating it routinely in stress reports.

## Supplementary Information

**Additional file 1. Supplement Table 1:** Neural network modelling to predict obstructive coronary artery disease.

## Data Availability

The data that support the findings of this study are available from the corresponding author upon reasonable request.
