# Supplementary material for: Clinical significance of the hemodynamic gain index in patients undergoing exercise stress testing and coronary computed tomography angiography
Source: BMC Cardiovasc Disord. 2023 Feb 3;23:65. doi: 10.1186/s12872-023-03088-z (PMC9898929; doi:10.1186/s12872-023-03088-z)
Supplement: Supplementary file 1 — Additional file 1. Supplement Table 1: Neural network modelling to predict obstructive coronary artery disease. [file 12872_2023_3088_MOESM1_ESM.docx]

**Supplement Table 1: Neural network modelling to predict obstructive coronary artery disease**

|  | **Model 1**  Adjusted for FRS, diabetes, BMI, DTS | **Model 2**  Adjusted for age, gender, HTN, DL, smoking, diabetes, BMI, DTS |
| --- | --- | --- |
|  |  |  |
| Post training % incorrect | 6.3% | 4.8% |
| Area under the curve | 0.830 | 0.955 |
| Normalized importance of HGI | 100% | 63% |
